# Meditation and interoception: a conceptual framework for the narrative and experiential self

**DOI:** 10.3389/fpsyg.2024.1393969

**Published:** 2024-10-16

**Authors:** Jonathan Earl Gibson

**Affiliations:** South Dakota School of Mines and Technology, Rapid City, SD, United States

**Keywords:** meditation, interoception, mindfulness, the experiential self, the narrative self

## Abstract

The concept of the self is complex and there is no consensus on what the self is. However, there are emerging patterns in the literature that point to two different selves, the narrative and experiential self. The narrative self refers to a conceptual or representational knowledge of the self that extends across time and manifests in self-reflection and personality assessments. The experiential self refers to first-person perception, moment-to-moment awareness, embodiment, and a sense of agency. These two selves are reliably linked to two distinct neural circuits, the default mode network (DMN) and the insula and salience network (SN). One of the consistent themes in the meditative and mindfulness literature is a change in the perspective of the self. In this paper, I will review how meditation alters those neural circuits providing a plausible mechanism that can explain the changes in the self. I also propose a rudimentary conceptual framework to account for some of the mixed results found throughout meditation literature.

## Introduction

The concept of the self is complex and difficult to define (Baumeister, 1991). There are a variety of ways in which people describe themselves including, but certainly not limited to, physical appearance, social roles, biographical details, belief systems both political and religious and so forth (Monti et al., 2022). The self is believed to be shaped by both cognitive and affective factors (Pelham, 1991) and many adults find it difficult to clearly articulate what their sense of self is (Bloom, 2007; Haidt et al., 2000). The self has also been conceptualized in varied ways within the scientific literature. Some have emphasized the self as the epicenter of knowledge, cognition, experience, and action (Varela et al., 1992). Some have emphasized the self’s multiplicity dividing it into different dimensions (Gallagher, 2000). Others have proposed a transpersonal perspective – that the self transcends the physical and temporal dimensions (Friedman, 1983; Mara et al., 2010), while others have argued that the self is ultimately an illusion (Gyamtso, 1994; Macy, 1991). Contemporary research indicates that the self is not an amalgamation of disconnected and unrelated aspects; those descriptions point to a unique individual (Monti et al., 2022).

The sense of self is also thought to evolve in concert with developmental milestones such as object permanence and theory of mind (Austin, 2000). Some have argued that the child’s sense of self is believed to emerge from distinguishing between psychological and biological states suggesting that the self is separate from the body which, for some, this separation holds true into adulthood (Anglin, 2014). In contrast, other studies have found that the self seems almost inseparable from the body (Seth, 2013; Monti et al., 2022; Hanley et al., 2021). Recent studies have found that both children and adults find it relatively intuitive locating the sense of self in the body (Hanley et al., 2021). Some have argued that that the sense of self or seat of self-awareness is ‘not just embodied, but deeply embodied’ (Monti et al., 2022) rather than embodiment as purely propositional (Schubert and Koole, 2009).

Recent advancements in neuroscience provide empirical evidence for the neurological functions of various altered states induced by meditation. These studies also provide an additional step toward understanding the developmental process of meditation, potential benefits, and possible detriments. Many spiritual or wisdom traditions (e.g., Buddhism, Vedic, Hindu practices, and Shamanism) include meditation in their practices. These traditions also include descriptions and insights into the nature of the self. However, there is a gap within the scientific literature in connecting these descriptions and insights with empirical data. In this paper I will review some of the meditation literature in hopes to provide a conceptual framework to help explain how meditation changes the perspective of the self. A fully comprehensive review on meditation and the nature of the self is beyond the scope of this paper, but I will highlight how meditation reliably alters distinct neural circuits consistently linked to different selves. Finally, I will make three proposals to help account for some of the mixed or inconsistent results in the mindfulness and meditation literature.

## Background: the narrative and experiential self

Despite the various ways in which the self has been conceptualized, much of the contemporary scientific research has divided the self into two broad categories: the narrative self (Gallagher, 2000) which includes the “me-self,” the object of perception (James, 1890) and the experiential self (Libby and Eibach, 2002) sometimes referred to as the pre-reflective or minimal self which includes the “I-self,” the subject of perception (James, 1890). The narrative self or “me” is a perception of the self that extends across time, accumulating conceptual or representational knowledge about the self. The narrative self manifests through self-reflection (Farb et al., 2007; Gallagher, 2013) and can be measured through personality assessments (McCrae and Costa, 1997). The “I” or experiential self refers to first-person perception, moment-to-moment awareness, embodiment, and a sense of agency (Farb et al., 2007; Seth, 2013; Monti et al., 2022; Nave et al., 2021). In short, the narrative self is the part of the self that can be observed or described (e.g., outgoing, friendly etc.) while the experiential self eludes observation and description because it is the part of the self that is doing the observing and describing.

### The narrative self and the default mode network

Recent studies have begun to reliably identify distinct neural circuits associated with these different selves. The Narrative or “me” self has been consistently linked to the default mode network (DMN) (Kelley et al., 2002; Northoff and Bermpohl, 2004; Northoff et al., 2006; Farb et al., 2007, 2013; Davey and Harrison, 2018; Lindström et al., 2023a), while the experiential self is consistently linked to the insula, the hub for interoception, and the salience network (SN) (Craig, 2002, 2009; Farb et al., 2007, 2013; Seth, 2013; Qin et al., 2020; Lindström et al., 2023a). Some of the preliminary studies investigating self-referential tasks found individuals recruit from a variety of cortical and subcortical structures. Both the preliminary and contemporary approaches identified anterior and posterior hubs within a cortical midline circuit, e.g., the DMN. The DMN is primarily composed of the medial prefrontal regions including the medial prefrontal cortex (mPFC), ventromedial prefrontal cortex (vmPFC), and dorsomedial prefrontal cortex (dmPFC). The posterior hub of the DMN includes the posterior cingulate cortex (PCC), the precuneus and the inferior and parietal temporal regions (Northoff and Bermpohl, 2004; Northoff et al., 2006; Zsadanyi et al., 2021; Cook et al., 2022; Lindström et al., 2023a). These hubs are linked to a variety of different functions that influence cognitive, affective, and behavioral responses to events (Davey and Harrison, 2018).

To begin, Northoff et al. (2006) found that self-referential processes associated with the DMN were not guided by cognitive or perceptual principles *per se*, but rather the meaning or investment of self-related stimuli. For instance, the vmPFC is believed to play a critical role in encoding the emotional importance and relevant quality of self-related aspects (Northoff et al., 2006). The dmPFC and PCC have both been linked to the reappraisal and analysis of self-related stimuli (Flagan and Beer, 2013; Zsadanyi et al., 2021). Other studies have linked the dmPFC and PCC to evaluation and certainty (Krain et al., 2006; Eldaief et al., 2011; Cooper et al., 2022) while the vmPFC has been linked to emotive importance of evaluations (Levy and Glimcher, 2011; Wlodarski and Dunbar, 2016). D'Argembeau (2013) suggested that the vmPFC plays a crucial role in connecting subjectively important stimuli to self-representations while others suggested that the dmPFC may play a broader role in mediating epistemic judgments (Lieberman et al., 2004). Brewer et al. (2013) found the PCC is associated with self-identifying features as ‘being a certain kind of person.’

Self-identifying features also assumes a working knowledge of the self across time (Neisser, 1997). Behavior regulation, for example, applies self-knowledge to determine if a given action conforms to one’s values and expectations of “who you are” (Spreng et al., 2009). Self-knowledge entails social, moral, and behavioral implications which are largely determined by one’s identity. Self-knowledge is also prone to various biases including self-enhancement (Brown, 1986) and self-verification motives (Swann, 2011). Many kinds or self-views are often rigidly upheld. Someone with a poor body image may stubbornly maintain a belief (“I am fat”) and cling to that notion despite contradictions from others or the environment. Fixed self-views are often upheld by the ‘stake’ or investment held in such claims (Pelham, 1991). That is, some might overidentify with certain components of the self and are thus jointly maintained by cognitive and affective processes. Pelham referred to these processes as epistemic and emotive self-view investments. Epistemic self-view refers to the degree of certainty one has, and emotive self-view refers to the emotional importance.

The DMN is distinctly associated with the maintenance of self-related capacities, including memory for self-traits (Kelley et al., 2002), reflected self-knowledge (Lieberman et al., 2004) and integration of self-referential stimuli in emotional and autobiographical context (Northoff and Bermpohl, 2004; Schmitz and Johnson, 2007). The DMN has also been found to be highly active during rest, mind wandering, and conditions of stimulus dependent thought which exerts a type of cognitive elaboration or narration of mental events associated with those functions (Northoff et al., 2006; Farb et al., 2007, 2013). Davey and Harrison (2018) wrote: “The role of the default mode network: as a dynamic entity that sums the activity of, and interaction between, other large-scale systems across the brain. The DMN acts to coordinate network integration to in-fluence the body’s response to events, thereby supporting flexible, adaptive behavior in complex environments. It is from this activity—which creates “a centre of narrative gravity”—that our sense of ourselves emerges” (pp. 3).

### The experiential self, interoception, and the salience network

In contrast, the experiential or minimal self seems to be more elusive and difficult to measure. Studies demonstrating increased body awareness or interoception plays a key role in integrating bodily processes that shape emotional experiences that are central to the self (Farb et al., 2007, 2013; Seth, 2013; Garfinkel et al., 2016; Babo-Rebelo et al., 2016, Monti et al., 2022; Matko et al., 2022). Early and modern theories alike have emphasized the importance of interoceptive feedback on affective states, cognitive processes, and behavioral patterns (James, 1890; Damasio et al., 1996; Cameron, 2001; Craig, 2002, 2009; Porges, 2011; Mayer, 2011; Seth, 2013; Blanke et al., 2015). Awareness of homeostatic functions such as heartbeat, hunger, thirst, gastric contractions, and other physiological processes seem to play a crucial role (Damasio, 2000; Critchley et al., 2004; Craig, 2009; Seth, 2013). Interoceptive sensations are sent to the insula, the hub for interoception, which are then translated into feelings and emotions which are believed to be crucial neuropsychological constructs that function as the currency of awareness (Craig, 2009). “In this view, the neural basis for awareness is the neural representation of the physiological condition of the body, and the homeostatic neural construct for a feeling from the body is the foundation for the encoding of all feelings” (pp. 66). Interoceptive awareness grounds us in the present moment and exerts a stabilizing influence on self-awareness (Cameron, 2001; Craig, 2009; Seth, 2013; Farb et al., 2015; Khalsa et al., 2018).

Integrating interoceptive processes is an ongoing, unfolding experience which is essential for relating to self, others, and the world (Damasio et al., 1996; Craig, 2002; Nave et al., 2021). This type of embodied awareness was central to Husserl’s and Merleau-Ponty’s phenomenology (Husserl, 1989). Merleau-Ponty (1962) argued that consciousness is fundamentally embodied. Attuning to this pre-reflective ontological state facilitates our experience and this embodied or corporeal awareness precedes reflective conscious thought (Merleau-Ponty, 1962, 1964). The body is not only the *object* of perception, but also the *subject* of perception (Marcel, 1952; Merleau-Ponty, 1962; Wertz, 1987). That is, we do not just have a body – we are bodily (Levin, 1985). Contemporary imaging studies are beginning to shed light on these processes.

Several theories provide a conceptual framework that illuminate how interoception is central to understanding the self (Damasio, 2000; Critchley et al., 2004; Craig, 2009; Seth, 2013). Hanley et al. (2021) report for most people children and adults alike, across cultures, identify the self in the same places in the body, primarily in the head and the heart. Studies exploring this concept (where people locate the self in the body) have found associations between self-location and personality, self-concept, decision-making, and emotion (Adam et al., 2015). The experiential self or “I” appears to be radically shaped by corporeal dimensions that can be assessed through interoception (Monti et al., 2022).

## Meditation and the self

One of the primary themes that have emerged in the meditative and mindfulness literature is these practices often lead to a change in the perspective of the self (Hölzel et al., 2011; Kerr et al., 2011; Gibson, 2014). Those who begin a mindfulness or meditation practice often describe a detachment from a static sense of self while no longer overidentifying with self-related concepts (Hölzel et al., 2011; Nave et al., 2021). That is, there is a general shift from the narrative self to the experiential self. In Buddhist philosophy, the first pillar to develop mindfulness is to develop a sense of the body, which includes an awareness of momentary sensation while distinguishing sensation from conceptual thought (Buddhaghosa, 2010). MacLean et al. (2010) found meditation improves clarity of thought and temporal resolution leading to a meta-awareness. Meta-awareness in this context refers to a form of subjective experience and executive monitoring as a distributed form of attention (Hölzel et al., 2011). This widened attentional state or meta-awareness seems to facilitate a detachment from a static or conceptual representation of self, i.e., the narrative self (Olendzki, 2006; Nave et al., 2021) or even leading to a deconstruction of the self (Epstein, 1988).

Attention regulation is the basis of all meditative techniques and is believed to be the prerequisite for other beneficial mechanisms to take place (Hölzel et al., 2011; Farb et al., 2015). Contemporary research has shown that attentional style in different meditations modulate different neural circuits which produce different effects on a wide range of cognitive and affective processes (Kok and Singer, 2017; Nave et al., 2021; Guidotti et al., 2023). These structural changes in brain networks are also associated with the perceptual shifts in the sense of self and in everyday experience (Lindström et al., 2023b). However, the literature is littered with mixed, inconsistent, and sometimes contradictory results. These differences are difficult to tease apart, and it is beyond the scope of this paper to fully do so. However, there are emerging patterns that are relevant to this thesis that will be covered below.

Despite the differences in the meditation and mindfulness literature, there are consistent, documented patters that have emerged relevant to the different selves, at least among a healthy population. The starting point for many novice mediators is typically a focused attention (FA) mediation (Lutz et al., 2008; Farb et al., 2015). The practitioner will focus on a chosen object, usually their breath or body sensation. Once practitioners begin to improve and stabilize their attentional control, there tends to be a gradual shift from an FA to an open monitoring (OM) or mindfulness technique (Lutz et al., 2008; Farb et al., 2015). The OM technique requires a different attentional style and produces different effects (Kok and Singer, 2017; Weng et al., 2020; Guidotti et al., 2023). The OM attentional style includes a broad, open, receptive awareness sometimes referred to as ‘awareness itself’ (Lutz et al., 2008). Focused attention meditations consist of a narrow window of attention while OM consists of a broad, open, receptive window of attention. Attentional style can be understood as a focal point from which to investigate complex interoceptive and exteroceptive sensations which inevitably shape cognitive and affective states influencing the perception of the self (Gibson, 2019).

## Meditation-induced changes

Kok and Singer (2017) conducted a study comparing four different mediations which are often included in a mindfulness practice. They covered two FA techniques (breathing and body scan mediations), an OM technique, and a loving-kindness technique. What they found was that certain meditations were slightly more effective at cultivating specific capacities and that each technique is characterized by its own distinct psychological experience. For instance, the body scan meditation led to greater improvements in interoception compared to the other three. The OM meditation led to greater improvement in meta-awareness. However, they highlight that all four mediations largely produced the same beneficial effects. Both the breathing and body scan mediations increased interoception and decreased mind wandering, but so did the loving kindness and OM mediations. Loving kindness increased ‘subjective warmth’, but so did the FA and OM techniques. They found that all participants, regardless of the meditative practice, increased in focus, interoceptive awareness, positivity of affect, warmth, and energy. Furthermore, and relevant to this paper, no practice significantly changed the amount of self-related thought. “All practices left participants feeling happier, more energized, more present in the moment, more aware of their bodies, and better able to disengage from distracting thoughts” (pp. 228).

Perhaps the most interesting finding from their study was that there were subtle, yet important differences between meditations but those differences seemed to depend on prior meditation experience. For example, they found improvements in meta-cognition or meta-awareness was linked to increased interoception. Once participants improved in interoception, then meta-awareness improved. Those who practiced the loving-kindness meditation without prior mediation experience, in this case the two FA techniques, never fully developed the same level of meta-awareness as the other participants. The authors suggested that some of the benefits from the loving-kindness meditation may be dependent upon increased interoception. The participants who were trained in the breathing or body scan techniques before learning the OM and loving-kindness techniques may represent a ‘carryover effect’ from the two FA techniques. Cultivating interoceptive awareness and present focus appears to be core features of meditative practice (Kok and Singer, 2017).

## The insula and salience network

Meditation produces numerous and diverse effects activating distinct neural circuits. For purposes of this paper there will be an emphasis on the insula, SN, and DMN as these are reliably linked to the different selves and are consistently and predictably modulated by meditation practice (see Fox and Cahn, 2021; Guidotti et al., 2023). The insula, perhaps due to its location, is still one of the least understood (Kortz and Lillehei, 2023) and underestimated (Namkung et al., 2017) regions of the brain, which is interesting given that it is the only region associated with *all* subjective experiences and human awareness (Craig, 2009). It is also the only region that is involved in and modulated by *all* meditations (Fox and Cahn, 2021). The insula is not only the hub for interoception but is essential for attention regulation (see Yu et al., 2022). The insula has reciprocal connections to the anterior cingulate cortex (ACC) of the SN and can facilitate cognitive control by acting as the switch between the Executive Control Network (ECN) and the DMN (Cole and Schneider, 2007; Sridharan et al., 2008; Menon and Uddin, 2010; Hasenkamp and Barsalou, 2012). The SN acts as a “cognitive control network” and forms a highly interconnected core system for task-dependent control of goal directed behavior and sensory processing (Brass and Haggard, 2007; Cole and Schneider, 2007; Dosenbach et al., 2007; Sharp et al., 2018). Interoceptive pathways have their primary cortical representation within the insula, but have bidirectional connectivity through numerous cortical and subcortical regions (Craig, 2009; Khalsa et al., 2018; Sharp et al., 2018; Yu et al., 2022). Given that attention regulation in meditation appears to be the prerequisite for other beneficial mechanisms to take place, the insula and SN ought to be emphasized as this network plays a critical role in these processes.

The insula also integrates information from the body via lamina 1 spinothalamic and vagal afferent tracts (Craig, 2002). Much of these body sensations projects ultimately into the posterior (pINS) portion of the insula and somatosensory cortices and is re-represented in the mid and anterior (aINS) portion of the insula (Craig, 2009; Farb et al., 2013; Seth, 2013; Khalsa et al., 2018; Yu et al., 2022). The aINS provides a multilevel integrated meta-representation of the state of the entire body integrating body sensations and exteroceptive stimuli into a broader context (Craig, 2009; Farb et al., 2013; Damasio and Carvalho, 2013; Yu et al., 2022; Wu et al., 2023). Afferent sensory signals are integrated on multiple levels within the insula providing a set of interoceptive maps across different body systems (Seth, 2013; Khalsa et al., 2018). Several authors have proposed a posterior-to-anterior gradient whereby posterior regions of the insula support objective mappings of interoceptive signals, whereas the anterior supports secondary re-representations integrated with top-down processes (expectations, beliefs, conditioned responses, prior experiences etc.) leading to one’s subjective feeling state (Critchley et al., 2004; Craig, 2009; Seth, 2013; Damasio and Carvalho, 2013; Farb et al., 2015; Khalsa et al., 2018).

The insula and SN also support a wide range of cognitive and affective functions which inform and shape *all* subjective experiences and the only common feature among these functions is that they engage the awareness of the subject (see Craig, 2009). This enhanced awareness or meta-awareness is also a defining feature in the mindfulness literature. Therefore, given the insula and the SN’s role in interoception, attention, and its link to all subjective experiences, the finding that all meditations modulate the insula should be unsurprising.

## Neuroplasticity effects of meditation on the SN and DMN

Numerous studies have described how mindfulness and meditation specifically modulates the insula and SN (Farb et al., 2007, 2013; Paulus et al., 2011; Santarnecchi et al., 2014; Friedel et al., 2015; Doll et al., 2015; Young et al., 2017; Sharp et al., 2018; Weng et al., 2020; Yu et al., 2022; Guu et al., 2023). Numerous studies have also described how mindfulness and meditation specifically modulates the DMN (Ott et al., 2010; Kilpatrick et al., 2011; Hasenkamp and Barsalou, 2012; Doll et al., 2015; Weng et al., 2020; Zsadanyi et al., 2021; Wu et al., 2023; Kral et al., 2022; Cooper et al., 2022; Lindström et al., 2023a). In short, these studies demonstrate a general pattern of increased activity in the insula and SN and decreased activity in the DMN, especially compared to a resting state. Two of the more illuminating studies on this process and how it relates to the self was conducted by Farb and his colleagues (Farb et al., 2007, 2013). They found the narrative self was linked to the DMN and the left lateralized language network. This network exerts a cognitive elaboration of mental events associated with rumination and mind-wandering that they referred to as a narrative focus (NF). The insula and SN, in contrast, was shown to inhibit cognitive elaboration of a mental event in favor of attending to the present moment in a broad, open-monitoring sensing state, which they called experiential focus (EF). Experiential focus was characterized as present-centered, sensing what is occurring in one’s thoughts, feelings, and body state. The participants in the study were asked to engage in a NF activity which activated DMN by reading trait-related adjectives and reflect on what the adjective meant to them as a person. In the EF mode, participants were asked to monitor their moment-to-moment experience in response to those adjectives. The experimental group went through an 8-week mindfulness training (MT) using the Mindfulness-Based Stress Reduction (MBSR) program.

After the MT, the participants engaging EF not only deactivated DMN and left lateralized language network involved in linguistic related processes but also rewired those circuits. The authors also discovered that the insula and SN decoupled from the DMN while simultaneously increasing connectivity within the insula which led to a larger reduction in the mPFC activity. Without MT, participants were more likely to activate the DMN when engaging in EF. The authors then described two distinct modes of self-reference linked to those neural circuits: “(1) higher order self-reference characterized by neural processes supporting awareness of a self that extends across time and (2) more basic momentary self-reference characterized by neural changes supporting awareness of the psychological present. The latter, represented by evolutionary older neural regions, may represent a return to the *neural origins of identity*, in which self-awareness in each moment arises from the integration of basic interoceptive and exteroceptive bodily sensory processes” (pp. 319 emphasis mine). After MT, there was a gradual shift in self-referential processes toward a more objective, self-detached analysis rather than conceptual self-referential process associated with the DMN. Recent studies have referred to this detached, meta-awareness as perspectival ownership (Lindström et al., 2023a,b).

The second study conducted by Farb et al. (2013) discovered that the control group – participants who were untrained in MBSR – when asked to focus on internal or external sensations revealed a consistent pattern of dmPFC activation. This should be unsurprising because the DMN has connections to the subcortical regions that influence the perception of affective and visceral responses (Davey and Harrison, 2018). In contrast, those who were trained in the MBSR program showed, again, reduced activity in the dmPFC and increased connectivity between the posterior and anterior insula leading to greater insula activation. Mindfulness meditation simultaneously reduces dmPFC and DMN activity and strengthens the inverse connection between the dmPFC and insula leading to a departure from mind-wandering and a conceptual representation of the internal state of the body to a more expansive and diffuse form of sensory attention. This process seemed to have a noted shift on the perspective of the self. The authors highlight that when the dmPFC, when active for executive functions, seems to suppress or limit unintentional interoceptive signals. Both trained and untrained populations in MT were able to activate interoceptive signals from the posterior to anterior insula when focusing on their breath (FA technique); the difference was that mindfulness trained participants appeared to possess this increased connectivity by default. The meditation process re-wired those circuits leading to enhanced connectivity within the insula while deactivating the DMN which provided the individual with a consistent “online” representation of the internal state of the body.

The dmPFC processes higher-order cognitions and relays that information to the anterior portion of the insula (aINS) as part of a top-down, evaluative process (Farb et al., 2013, 2015). Focused attention on the body (i.e., meditation) dampens dmPFC activity causing the aINS to attend more fully to the incoming internal stimuli being sent to the pINS from the body leading to a neuroplasticity change in the posterior, mid, and anterior insula (Damasio and Carvalho, 2013; Farb et al., 2013; Yu et al., 2022) while simultaneously decoupling the insula from the DMN (Farb et al., 2007, 2013; Doll et al., 2015). Many studies have since confirmed this pattern (Kilpatrick et al., 2011; Hasenkamp and Barsalou, 2012; Doll et al., 2015; Sharp et al., 2018; Weng et al., 2020; Yu et al., 2022; Guu et al., 2023; Guidotti et al., 2023). For instance, Weng et al. (2020) found that focused attention on external stimuli and internal sensations yielded distinct neural signatures and participants who were able to focus on their breath (interoceptive stimuli) over a longer period of time were able to dampen self-referential processing linked to the DMN (see also Doll et al., 2015).

As previously mentioned, it is important to note that there are mixed and sometimes contradictory results in the literature some of which will be briefly outlined below. In general, however, the pattern of increased activation and connection within the insula and SN, decreased connection between the SN and DMN, and decreased connection and activation within the DMN is well documented. These alterations can provide the mechanism that can explain the gradual changes of the sense of self after a meditation practice. In the absence of cognitive evaluation, judgment, or rumination cognition may be freed to consider alternative interpretations of the internal states (Farb et al., 2015). Doll et al. (2015) wrote: “The present data might suggest that more mindful individuals may have a lower correlation between these networks, which could indicate an increased switching of attention away from self-related towards, e.g., more sensory focused processing” (pp. 7). Enhanced interoceptive awareness grounds us in the present moment and exerts a stabilizing influence on self-awareness (Craig, 2009; Seth, 2013; Farb et al., 2015; Khalsa et al., 2018). Studies have shown that interoceptive awareness and emotion regulation is often associated with top-down strategies in novice meditators, but long-term or practiced meditators tend to use bottom-up strategies by integrating interoceptive information into broader conscious states (Chiesa et al., 2013; Farb et al., 2007, 2013). These brain-activity signatures have particular relevance to self and non-self referential states that advanced meditators can attain (Nave et al., 2021; Yang et al., 2024).

In a mixed methods study (Gibson, 2014), participants practicing two non-mindful, FA techniques (breathing and body scan meditation) would often organically journal and describe these types of changes. One participant wrote: “I became aware of something today, a feeling of resistance to looking into myself… I hope to further use these techniques to aide me in letting go of my resistance and become more aware of the real me” (pp. 187). Another wrote: “It [body scan] helps me notice quirks or stress in my body and also where my pent-up emotions are weighing on me. It gives my body a voice when normally it does not have one. That “voice” has always been there, I am just becoming aware of it” (pp. 189). Another wrote: “The meditations helped me tap into myself. Be more aware of what’s going on in my own mind. When I was younger my parents would ask “what’s wrong”? But now I am able to express myself better. I am more aware of what’s going on in my mind, how I am feeling, why I did what I did” (pp. 429). Another wrote: “The body-scan helps with knowing who you truly are. It gets into the unconscious and brings it into the conscious. You not only find what is happening with you physically, but also emotionally” (pp. 183).

## Gaps in the literature: meditation and large-scale brain networks

Some recent studies have investigated whole-brain or large-scale brain network topology after mindfulness and meditation training. Whether mindfulness and other meditations primarily impacts brain function locally or dynamics of large-scale brain networks remains unclear (Guu et al., 2023). For example, Sharp et al. (2018) found ‘significant increases in connection strength in the right insula across all of its connections,’ but no global changes in whole-brain analysis. Other studies have found large-scale brain alterations. For example, Josipovic et al. (2012) found increased connectivity between the DMN and fronto parietal network FPN. Others found increased connectivity between the DMN, FPN, and SN (Hasenkamp and Barsalou, 2012). Guu et al. (2023) found enhanced connectivity between the SN and ECN, but the alterations were dependent upon different meditations practiced. There was increased connectivity between the SN and the occipital region with focused breathing (a FA technique), while body-scan (another FA technique) increased SN connectivity to the frontal/central gyri and parietal lobe during a resting state. They also discovered that the two FA meditations produced different effects within the insula and SN at rest, but also noted increased activity and a global enhancement of the SN during these ‘mindfulness’ meditations.

Yu et al. (2022) investigated how meditation modulates distinct subregions within the insula while others have examined specific functions within the SN (Guu et al., 2023). There is evidence that the insula has a much stronger association with interoception but a weaker association with cognitive control whereas the ACC showed the opposite pattern (see also Craig, 2009; Sharp et al., 2018). Similarly, Brewer et al. (2011) found that expert meditators have novel connections between the ACC and the dlPFC of the ECN leading what they linked to enhanced attentional control, but these changes may be dependent upon meditation type. In this case, these connections were due to an OM type meditation as opposed to a loving kindness meditation. Kral et al. (2022) also found that practiced meditators have a stronger connection between the ACC and dlPFC in conjunction with decreased connection strength across the DMN hubs compared to meditative-naïve controls. The expert meditators were practiced in either a Vipassana and, in contrast to Brewer et al. (2011), loving-kindness meditation. Some studies have shown that anterior and left-lateralized regions of the DMN is linked to the narrative self, while right lateralized areas of the DMN are linked to the experiential self (Fingelkurts et al., 2022) Qin et al. (2020) found that multiple regions are involved in the narrative self, including the DMN, SN, thalamus, superior frontal gyrus, right premotor cortex, and temporal parietal junction (TPJ).

Guidotti et al. (2023) found that both FA and OM meditation altered 10 distinct networks among expert meditators. The most relevant connections were linked to the SN and DMN. In contrast to other studies, the least relevant connections were found in the right ECN and sensorimotor networks. The authors found numerous alterations and connections across and within each hemisphere. Interestingly, like Farb et al. (2007, 2013), they found the language network was also modulated by meditation which has a robust connection to the DMN. One of the unique contributions from this recent study was a focus on the specific effects of different meditations on distinct neural circuits. Despite the unique findings and documented differences, the authors found the most relevant networks shaped by meditation were the SN and DMN. Similarly, Qin et al. (2020) found one of the major themes that emerged from their study was the ‘common involvement’ of the insula in three different self-differentiation processes. They found the insula and interoception plays a critical role in linking various networks in the organization of mental-self-processing.

## Future directions: three proposals

Meditation has shown to consistently alter the insula, SN, and DMN. Yet, much is still unknown on how different meditations modulate different networks and produce varied neurological and phenomenological effects. For the purposes of this paper, I hope to provide a rudimentary framework in the hopes of clarifying some of the confusion and orient future research. I submit that it will prove fruitful to 1) distinguish and specify between different meditations and include meditation experience to explore their individual effects; 2) clearly differentiate between mindfulness and interoception as these are two similar yet distinct mind–body constructs and teasing those apart should help clarify some of the confusion; and 3) consider and incorporate individual practitioner histories through the lens of early life experiences, trauma, and the attachment relationship. There is mounting data clearly indicating how early life experiences and childhood trauma (CT) have been reliably shown to alter the development and function of numerous neural circuits, including the SN and DMN (Oldroyd et al., 2019; Ireton et al., 2024).

### Proposal one: distinguish between meditation type and experience

Despite the mixed results within the mindfulness and meditation literature, the alterations in the SN and DMN among a healthy population are reasonably consistent (see Hölzel et al., 2011; Kok and Singer, 2017; Gibson, 2019; Nave et al., 2021; Guidotti et al., 2023). Mindfulness and all meditations reliably alter the insula and SN which, in turn, enhance interoception with its associated functions and benefits (Farb et al., 2015; Kok and Singer, 2017; Hanley et al., 2017; Mehling et al., 2017; Sharp et al., 2018; Yu et al., 2022; Guu et al., 2023). Guidotti et al. (2023) demonstrated that meditation type or attentional style plays a critical role in shaping various networks typically included in the meditation literature. For example, when studying the effects of both the FA and OM techniques, they found that the ECN, which is classically defined as a core system for coordinating attentional processes, seems to be ‘less prone to predict meditation style’ while the SN and DMN were consistently involved and easier to predict. In other words, expert meditators seem to produce consistent, large-scale alterations which they referred to as ‘style-specific fingerprints.’ The authors found it much easier to predict these specific large-scale ‘fingerprints’ in the expert group compared to the novice group indicating that long-term meditation practices reliably modulate specific pathways in the SN and DMN. With this basic premise, future research can explore the effects of different meditations through meditation type and experience. It is important to note that the identified consistent or predictable results may only occur in a healthy population. This issue will be addressed further below.

### Proposal two: mindfulness or interoception

Notwithstanding its growing popularity in the scientific community and society generally, mindfulness itself remains broadly defined, loosely conceptualized, and poorly understood (Van Dam et al., 2017; Gibson, 2019; Grossman, 2019). Van Dam et al. (2017) argued that mindfulness has become an umbrella term that characterizes a large number of practices, processes, and characteristics spanning acceptance, awareness, non-judgment and memory. The semantic ambiguity in the meaning of mindfulness or mindful meditations has implications and the authors urged scientists, practitioners, and the media alike to move away from the broad use of the term mindfulness and explicitly describe which practices and processes are being taught. A mindfulness meditation is an ‘open monitoring’ (OM) meditation which explicitly prescribes a mindful attentional style to both interoceptive and exteroceptive sensations, thoughts, and emotions (Raffone and Srinivasan, 2010; Fissler et al., 2016). However, FA meditations are consistently included in mindfulness practices which can be problematic because meditations require different attentional styles which activates different neural circuits and produces different psychological effects (Kok and Singer, 2017; Weng et al., 2020; Fox and Cahn, 2021; Nave et al., 2021; Guidotti et al., 2023). The mindfulness-based stress reduction (MBSR) program, for example, consists of multicomponent treatments and employs both FA meditative techniques (body scan and yoga) and an OM or mindfulness technique (sitting meditation). Yet, all these techniques require different attentional styles “making it impossible to isolate” the effects of any one specific practice (Kok and Singer, 2017) and it is still unclear what role ‘mindfulness’ may play (Hölzel et al., 2011).

There seems to be a growing trend in some of the literature focusing on specific meditations and observing their individual effects (see Guidotti et al., 2023; Yang et al., 2024). However, other recent studies are still referring to FA meditations as a mindfulness mediation (see Wu et al., 2023). A prevailing assumption is that many of the benefits and changes identified in the literature, including a change in the perspective of the self, are due to a ‘mindfulness’ practice, however it is operationalized (Hölzel et al., 2011; Doll et al., 2015; Sharp et al., 2018; Yu et al., 2022; Wu et al., 2023). But is this the case and is this the most accurate framework? Mindfulness meditation is an attentional style. It is how one attends to the present moment which often includes an open, objective, non-reactive, non-judgmental, open-heart (Kabat-Zinn, 2013). But in praxis and described throughout the literature, this attentional style does not often differentiate between interoceptive and exteroceptive sensations, thoughts, or emotions (Mehling et al., 2017; Hanley et al., 2017). Attention regulation is the foundation for all mediation practice. How and where one focuses attention is important and the broadly defined ‘mindfulness’ practices still leave room for interpretation and confusion (Van Dam et al., 2017; Gibson, 2019).

A growing belief among many researchers is that interoception is foundational to developing mindfulness (Farb et al., 2015; Hanley et al., 2017; Mehling et al., 2017; Sharp et al., 2018; Guu et al., 2023). Studies clearly show that the insula is associated with greater dispositional mindfulness (Creswell et al., 2007; Murakami et al., 2012; Haase et al., 2016; Yu et al., 2022), and mindfulness is consistently linked with increased insular volume and activity, especially in the aINS (see Wu et al., 2023; Guidotti et al., 2023 for review). However, ‘non-mindful’ contemplative practices such as yoga or yoga-based practices (Froeliger et al., 2012; Villemure et al., 2014; Schmalzl et al., 2015; Matko et al., 2022), non-mindful FA meditations (Gibson, 2014), and tai chi (Kerr et al., 2008) increase interoception and produce similar effects by simply attending to the body without specifically attending to the body in a traditionally mindful way, i.e., non-judgmental, non-reactive, open-hearted (see Kok and Singer, 2017). The insula provides distinct functions clearly relevant to mindfulness with a ‘measurable neurobiological imprint’ (Friedel et al., 2015). Friedel and his colleagues make the following argument: “While evidence for anterior insula involvement in adult long-term meditator has been interpreted to indicate an effect of mindfulness meditation on insula structure and function, the current results suggest that structural development of the anterior insula may contribute to the development of dispositional mindfulness” (pp. 67).

Attentional control, acceptance, interoception, emotion awareness and regulation are linked to mindfulness practices and insular function (Sharp et al., 2018; Yu et al., 2022; Wu et al., 2023). The aINS maintains attentional control which enables moment-to-moment awareness by providing an integrated representation of the present moment by sensing, interpreting, integrating, and regulating interoceptive processes and top-down cognitive processes into a larger context (Craig, 2009; Farb et al., 2015; Khalsa et al., 2018; Yu et al., 2022). Perhaps part of the confusion surrounding these constructs and their effects may be due to mislabeling. Is it an open, non-reactive, non-judgmental attentional style producing these effects? Or is it increased awareness of and an enhanced ability to integrate those processes into higher states of consciousness? It has been suggested that it is the latter (Gibson, 2019), but future research ought to disentangle these constructs for further clarity.

### Proposal three: early-life experiences, trauma, and attachment in the interpretive framework

Childhood trauma (CT) has enduring consequences on critical neural circuits throughout the lifespan (Raby et al., 2017; Fraley and Roisman, 2018). There is also a well-established relationship between CT and insecure attachments (Stevenson et al., 2021; Roters and Book, 2024) and insecure attachment and interoception (Oldroyd et al., 2019), mindfulness (Boughner et al., 2016), and psychological disorders (see Zsadanyi et al., 2021). The insula and SN show protracted post-natal development (Oldroyd et al., 2019). The architecture and function of those neural circuits are heavily shaped by early experiences and relationships. Some have even argued that normal brain development may be dependent upon a secure attachment (Schore, 2000) which is characterized by sensitive, loving, and supportive relationships (Ainsworth et al., 2015).

Investigating CT from the attachment relationship can help clarify how these early life experiences directly shape interoceptive capacities and its associated functions, including a healthy, developed sense of self. There is growing evidence that interoception develops initially in the attachment relationship and attachment related processes have been linked to insular anatomy and activity (see Oldroyd et al., 2019). A child’s attachment relationship characterized by either a warm and responsive connection with the primary caregiver, or a distressing relationship characterized by trauma, neglect, or indifference inevitably shapes the development of those neural circuits. Those with an avoidant or anxious attachment pattern have markedly lower insular volume and smaller surface areas than those with a secure attachment (Sheffield et al., 2013; Lim et al., 2014; Klabunde et al., 2017; Oldroyd et al., 2019). Furthermore, those with avoidant attachment patterns have decreased insular activity and maladaptive alterations within the SN. Studies show that the ACC does not fully connect with the aINS in those with an insecure avoidant attachment leading to blunted emotional affect (Lim et al., 2014; Oldroyd et al., 2019). In short, CT affects both the strength of interoceptive signals and how those signals are perceived (Oldroyd et al., 2019; Ireton et al., 2024).

Victims of CT struggle to accurately process interoceptive sensations which, in turn, disrupts their ability to connect to themselves and others (Oldroyd et al., 2019; Ireton et al., 2024). Hyper-arousal and hypo-arousal are two common response patterns to trauma (Ogden et al., 2006; Porges, 2011; Beydoun and Mehling, 2023). Hyper-arousal and hypo-arousal are also linked to anxious and avoidant attachments, respectively (Oldroyd et al., 2019; Ireton, et al., 2024). It is not uncommon for a person with a history of abuse or trauma to be overcome with anxiety to the point that it is impossible to experience or notice positive interoceptive sensations (Farb et al., 2015; Beydoun and Mehling, 2023). Hogeveen and Grafman (2021) found alexithymia is a common result of CT and a key factor responsible for non-adaptive strategies for regulating emotions. Alexithymia is defined as an impaired ability to be aware of, explicitly identify, and describe one’s feelings (Hogeveen and Grafman, 2021). The authors argued further that insecure attachments are the strongest predictor for developing alexithymia: “avoidant attachment style has the strongest negative impact on the development of a strategy for affect regulation and general emotional development” [pp. 9]. There is an extensive body of literature that has linked CT and insecure attachments to alexithymia (see Zdankiewicz-Ścigała and Ścigała, 2018).

The effects of trauma can lead to fragmentation and disconnection from one’s thoughts, emotions, body sensations, and oneself (Ogden et al., 2006; Herman, 2015). “To the extent that a child’s bodily experiences are denied, devalued, ignored, or punished by parents, the child will find ways to avoid feeling them, and develop a distorted sense of interoception” (Oldroyd et al., 2019, pp. 10). There is ample evidence that poor interoception is also linked to mental health disorders (Garfinkel and Critchley, 2013; Khalsa et al., 2018; Zamariola et al., 2019; Nord et al., 2021) and is a major contributor to emotional reactivity (Rae et al., 2019). Farb et al. (2015) argued that for some there may be an absence of interoceptive information and in that absence fears or anxiety may take on a quality of rumination and rumination is consistently linked to the DMN. Furthermore, even when a child is not thinking about the traumatic experience, victims struggle to accurately process interoceptive information (Ireton et al., 2024). Rumination may prevent the individual from accurately detecting interoceptive processes and integrating those into a broader psychological state. For instance, the dmPFC has been shown to suppress interoceptive information which might also explain how rumination can often dominate attention (Farb et al., 2013).

The aINS is also associated with elevated anxiety levels or hyper-arousal (Terasawa et al., 2013; Mallorquí-Bagué et al., 2014). As Porges (2011) points out, traumatized individuals usually have an abnormally active insula. The insula has been shown to have a meta-memory function in comparing feelings in the present moment with those of the past and anticipation of the future (Preuschoff et al., 2008). This meta-memory process can explain how both hyper-arousal and hypo-arousal lead to altered interoceptive predictions. Meta-memory functions of interoceptive predictions, sometimes referred to as predictive coding (PC), associated with trauma are often distorted (Paulus et al., 2011). Trauma may condition the autonomic nervous system or create a mismatch between actual physiological bottom-up interoceptive states and top-down perceptions or mental representations of those states.

## Meditation, interoception, and predictive coding

Interoceptive inference or PC is the view that prediction and error correction provide a fundamental principle for understanding brain processes. Predictive coding believed to be the result of probabilistic knowledge driven inferences from external and internal sensory signals (Seth, 2013). Predictive coding is also linked to a subjective sense of self which includes integrating homeostatic processes, the sense of owning and identifying with a body, first-person perspective, intention and agency, metacognitive aspects that are linked to the experiential self (Seth, 2013; Apps and Tsakiris, 2013). The aINS plays a central role in these processes (Seth, 2013). Seth argued a healthy or accurate PC framework is linked to unifying mechanisms of self-representation ‘what is me’ and ‘what is not me’ by integrating interoceptive processes. With rich connections throughout the brain, the aINS is structurally situated to process internal and external perceptions and work as both a comparator registering top-down and bottom-up processes with future prediction capacities (Apps and Tsakiris, 2013; Seth, 2013; Khalsa et al., 2018). An individual with a well-developed and healthy PC can fluidly integrate bottom up and top-down signals to reduce the mismatch of actual and expected states which would minimize ‘prediction error’ (Seth, 2013; Sharp et al., 2018).

In similar fashion, a new contemporary model, the psychosomatic intelligence hypothesis (PI-hypothesis), assumes and measures the impact of prior experiences on somatic signaling that influences body and self-regulatory behavior which shapes knowledge about the self (Fazekas et al., 2022). Those who score higher in this scale are more successful in detecting, interpreting, and integrating interoceptive signals in a healthy, self-regulatory manner (Fazekas et al., 2022). Meditation can enhance or clarify interoceptive signaling, but there are individual differences in ability to both generate and perceive subtle bodily changes (Zaki et al., 2012). Early life experiences, CT, and insecure attachment attachments seem to disrupt abilities to accurately detect interoceptive signaling which ultimately influences the sense of self. Therefore, it should prove fruitful to incorporate individual histories through the lens of early life experiences, including trauma and the attachment relationship in understanding these developments.

## Trauma, meditation, interoception, and the self

The effects of trauma on the nervous system are widely discussed in the literature. Research has clearly demonstrated that early life experiences, including CT have enduring consequences throughout the lifespan and alters numerous brain networks including the hypothalamus-pituitary–adrenal (HPA) axis (Bush et al., 2011; Palmer et al., 2013), the SN (Lim et al., 2014; Klabunde et al., 2017; Oldroyd et al., 2019), and the DMN and ECN (Ireton et al., 2024). The alterations to these circuits affect stress and emotional regulation (Raby et al., 2017; Fraley and Roisman, 2018), interoception (Oldroyd et al., 2019), mindfulness (Boughner et al., 2016; Gibson, 2024), and is linked to a large number of psychological disorders (Chen et al., 2022) which all have noted effects on the sense of self. Indeed, studies show that CT can alter one’s identity and sense of self (Ogden et al., 2006; Beydoun and Mehling, 2023 for review) by disrupting neural circuits and internal working models linked to interoception and emotional regulation (Oldroyd et al., 2019; Ireton et al., 2024). These disruptions in the internal working models and alterations in brain development can impede the ability to accurately discern sensations that shape emotions and thoughts (Vazire, 2010; Ireton et al., 2024). A recent meta-analysis found how chronically unmet needs in childhood led to defective or maladaptive schemata in adulthood (Bishop et al., 2022). Beliefs such as “I am a failure,” or “I am worthless and unlovable,” were the biggest cognitive risk factors for depression. Maladaptive self-views are often resistant to change either through direct feedback (Vazire et al., 2010) or introspection (Pronin, 2009).

Self-related PC engages multiple levels of self-representation and integrates multiple dimensions and subjective feeling states (Seth, 2013). Childhood trauma can create a maladaptive and unhealthy PC framework and alter critical neural circuits supporting those perceptions. Meditation has been shown to reliably alters those circuits (the SN and DMN); therefore, meditation may help some address maladaptive schemata. For example, Farb et al. (2010) found the DMN is linked to cognitive elaboration, increased self-focus, and rumination as part of a reappraisal process. They also found elevated levels of sadness is linked to activation in the DMN and deactivation of the SN and adjacent regions. The PCC of the DMN is involved with self-representation and being “caught up” or attached to identifying features of the self, “being a certain kind of person” (Brewer et al., 2013). Therefore, some may lack the perspective to objectively recognize investments in particular maladaptive self-views and may over identify with those schemata shaped by early life experiences. Meditation “strongly alters the DMN, mostly in a negative direction” (Lindström et al., 2023a) and decreased activity in the PCC is one of the most robust findings in the meditation literature (Cooper et al., 2022). The DMN is distinctly associated with the maintenance of self-related capacities including memory of self-traits (Kelley et al., 2002), reflected self-knowledge (Lieberman et al., 2004) and integration of self-referential stimuli in emotional and autobiographical context (Northoff and Bermpohl, 2004; Schmitz and Johnson, 2007).

## Detrimental effects of meditation and plausible alternatives to enhance interoception

Despite many of its benefits, meditation may not be an appropriate intervention or practice for some. Meditation has been shown to trigger autonomic hyperarousal, perceptual disturbances, traumatic memory re-experiencing, psychosis, and relaxation-induced panic (see Van Dam et al., 2017). Given that CT alters the development, structure, and function, of the insula and SN, one can see how meditation could potentially elicit past trauma (Porges, 2011; Seth, 2013), insecure attachment pattens (Oldroyd et al., 2019), and maladaptive PC schemata embedded in those circuits that might be too aversive to be accepted or controlled (Farb et al., 2015). Moreover, dysfunctions within the insula are linked to a higher degree of prediction errors, poor interoception, and emotion dysregulation (Seth, 2013). Several studies have identified disruptions in the dorsal mid-insula across a number of psychological disorders that were found to be anatomically distinct from other brain regions in affective processing (Uddin, 2015; Nord et al., 2021). Trauma fundamentally alters interoception, and thus alters one’s sense of self. There may be safer or more effective ways to foster interoception for some individuals which may include other body-centered practices such as yoga, tai chi, exercise, or even biofeedback training.

Another approach to enhance interoception could include investigating attachment and relational patterns. Recent studies have shown that attachment orientation develops first and can predict and affect the capacity for interoception and mindfulness in adulthood (Boughner et al., 2016; Stevenson et al., 2021; Beydoun and Mehling, 2023; Gibson, 2024). Roters and Book (2024) found that Insecure attachments linked to abuse, neglect, and disordered personality traits can “present as stronger symptomology than mindfulness traits.” If interoception develops initially in the attachment relationship (Oldroyd et al., 2019), then healthy relationships may help cultivate and enhance accurate interoception. Indeed, there is evidence that interoception is central to relational connections (Arnold et al., 2019). Another option may include psychedelic assisted psychotherapy as there is growing evidence for the efficacy of psychedelics treatments. One interesting correlation between psychedelic treatment and meditation is both have been shown to alleviate symptoms of psychopathology and lead to alterations in the sense of self (Millière et al., 2018). Both meditation and many psychedelic compounds produce similar neurological effects by specifically deactivating and desynchronizing the DMN (Siegel et al., 2024) and activating the SN (Walpola et al., 2017).

## A mindful attentional style to interoceptive processes

For many, however, the ability to attend to and maintain attention (i.e., meditation) on interoceptive sensations can disrupt automatic habitual responses to stress and facilitate more adaptive regulatory strategies which can enable one to more easily focus on specific interoceptive signals and integrate those into a broader contextual representation (Farb et al., 2015). Increased access to interoceptive information may also provide a richer set of data from which to investigate the relationship between habitual response, interoceptive sensations, top-down perceptions, and internal working models (Seth, 2013; Farb et al., 2015). Indeed, there is growing evidence that the ability to maintain attention on conditioned responses appears to be necessary for successful extinction of conditioned responses (Hölzel et al., 2011; Mehling et al., 2017). Therefore, bringing a widened or objective attentional style to interoceptive sensations may be a healthy and adaptive practice for a clinical population (Hanley et al., 2017). Mehling et al. (2017) point out, being able to mindfully accept body sensations may reduce the emotional impact of unpleasant ones. This capacity may also enable one to ‘listen’ to emotion-related sensations that are central to insight and decision making rather than being ‘overrun’ by them (Mehling et al., 2017; Hanley et al., 2017).

Effective emotion regulation involves different regulatory strategies, including non-reactivity while observing internal sensations and emotions which is believed to be done in part by refraining from any appraisal process (Chambers et al., 2009; Farb et al., 2010). Emotional awareness, emotional regulation, and accurate interoception can be enhanced by focusing on and refraining from evaluating sensations that have yet to be transformed through judgment (Farb et al., 2015; Matko et al., 2022). Critchley et al. (2004) suggested that reduced activation in the insula (which can include alterations in PC) during a negative emotional state is associated with accurate interoception. Simply, the ability to develop meta-awareness or a wider attentional frame may help one tolerate and integrate negative affect (Farb et al., 2010). Thus, intentional mindful awareness may help cultivate a safe focal point from which one can view various signals from the body leading to accurate interoception and an integrated sense of self (Gibson, 2019). Friedel et al. (2015) argue that there should be increased emphasis on the insula and SN network as these circuits not only play a critical role in maintaining emotion and self-regulation, but also provides a distinct construct with a measurable neurobiological imprint.

Studies have shown that increased or accurate interoception and meta-awareness of emotions is associated with reduced endorsements of dysfunctional schemata (Fresco et al., 2007; Frewen et al., 2007). Again, emotion regulation is often associated with top-down strategies in novice meditators, but long-term or practiced meditators tend to use bottom-up strategies to enhance and refine these signals to improve interoception (Chiesa et al., 2013; Farb et al., 2007, 2013). Farb et al. (2010) suggested that a mindful attentional style to interoceptive sensations can reduce emotional interference enabling one to view interoceptive signals and emotions as innocuous sensory information rather than an affect-laden threat to the self. “Disengaging reappraisal of negative affective content, in favor of engaging attention toward sensory integration, would allow for the generation of novel affective appraisals rather than attempting to manipulate an existing negative cognitive set” (pp. 10).

## Summary: meditation, experience, and attention style

Due to the complex, multidimensional structure of the self, it should be unsurprising that meditation can lead to diverse and expansive results. Even among practiced meditators, there are noticeable differences in both brain activity and phenomenological experience. For instance, Lindström et al. (2023a,b) explored how meditation shapes the sense of self through the framework of boundedness—as in being in one’s body, and boundarylessness—as the perspective of being in everywhere or everything. There is a lot of rich descriptive and imaging data in those studies, and I would invite the reader to review those for reference. For the purposes of this paper, a similar and important theme emerged: the authors suggest these two states (boundedness and boundarylessness) appear to share the neural underpinnings of “embodiment.” When participants were asked to ‘focus on the centre of their experience’ they found decreased activation in the main DMN hubs, and increased activation in the insula, thalamus, and BA6 region (see also Qin et al., 2020). The aINS, premotor cortex, and the right supplementary motor was also involved in a sense of agency. The sense of agency and being an observer is central to the experiential self and has been linked to insular and SN function for years (Craig, 2002).

Lindström et al. (2023a,b) data corroborated prior studies and concluded that meditation experience leads to varied outcomes (Gibson, 2014; Kok and Singer, 2017; Guidotti et al., 2023). Different meditations led to alterations in self-descriptions but, importantly, those alterations seemed dependent upon prior meditation experience. The authors reported those who described the highest levels of meta-awareness or what they described as perspectival ownership (PO) – a detached witness – found a decrease in DMN activity. Perspectival ownership, the subjective ‘I’, is understood as a core component of the experiential or pre-reflective self (Lindström et al., 2023b). Indeed, some of their participants described PO as an “observing self” or “real self” and the meditative practice became a journey of discovering their “inner observer.”

Nave et al. (2021) also conducted an in-depth phenomenological analysis exploring how meditation alters the sense of self or self-boundaries (SB). Self-boundaries were defined as a dynamical self and non-self distinction separating the ‘lived body’ from the external environment. There were noted differences between the participants in both the Lindstrom and Nave studies. Nave and his colleagues studied practiced meditators while Lindstrom and her colleagues studied individuals from a wide range of meditation experience, including beginners. Both studies, however, found attentional style and meditation practice and experience shaped the outcome. For instance, practiced meditators when engaged in a process of ‘letting go’ widened their attentional style and altered their sense of agency. This process led to a self-transcendence and global dissolution of the self (Nave et al., 2021). In contrast to the process of “letting go,” the authors found that a body scan meditation led to a clearer perception of distinct body boundaries. Some participants found that FA techniques reified the perception of the practitioner as an active, embodied agent controlling mental activity, while others identified their self with the expanding boundaries of attention, neglecting the body all together. These results emphasize the range of self-boundary flexibility and individual variation. Practiced meditators can profoundly change their sense of self while meditating.

## Commonalities

Despite the differences among practitioners, similar themes continue to emerge. Nave et al. (2021) identified six thematic structures developed among practiced meditators: 1) sense of location, 2) agency, 3) first-person perspective, 4) attention, 5) body sensations, and 6) affective valence or tone. All six themes have all been linked to the experiential or minimal self, the insula and SN. Like Husserl (1989) and Merleau-Ponty (1962), they argue that the fundamental aspect of the sense of self is its pre-reflective dimension as an embodied knower and agent and the relationships between these categories revealed a unitary dimension giving rise to the basic sense of being a bounded self (Nave et al., 2021). They wrote: “the pre-reflective self is not separate from the process of perceiving and acting” (pp. 27). Like Qin et al. (2020) they found the sense of self arises in concert with attention-demanding interactions within the environment. In other words, the sense of self seems to emerge in the integration between interoceptive and exteroceptive stimuli (see Seth, 2013; Farb et al., 2015; Khalsa et al., 2018; Qin et al., 2020), of which the aINS plays a unifying role. The insula and SN are believed to comprise the neural origins of identity (Farb et al., 2007) and the self appears to be embodied as both subject and object (Merleau-Ponty, 1962).

Nave et al. (2021) also emphasized attentional style as a key mechanism inherent in these processes. The attentional style inherent in different mediations can elucidate the role of self-inquiry and insight involved in the construction and deconstruction of the reflective and pre-reflective features of the self (Nave et al., 2021). They argued that attention is regarded as an active, self-regulatory process which changes how one engages with the self and world. McGilchrist (2012) argued that attention is not just another ‘function’ alongside other cognitive functions. Its ontological status precedes all other cognitive functions. “The kind of attention we bring to bear on the world changes the nature of the world we attend to, the very nature of the world in which those ‘functions’ would be carried out, and in which those ‘things’ would exist. Attention changes what kind of thing comes into being for us: in that way it changes the world” (pp. 28).

Attentional stye associated with deep states of self-dissolution not only radically alter the sense of self, but also shapes other non-self experiential structures, such as the surrounding space or external world (Qin et al., 2020; Nave et al., 2021). Quieting the mind and reducing self-invested conceptual representations and mind-wandering activity contributes to these sense-making mechanisms from which the self and world co-emerge. The central role of attentional engagement shapes the emergence of selfhood, and the data shows the aINS and increased interoception plays a fundamental role in these processes. Maintaining attentional control can be “especially useful in deconstructing the more persisting structures of self-experience” (Nave et al., 2021, pp. 23). Practiced meditators show consistent alterations in the SN and DMN leading to increased prediction accuracy (Guidotti et al., 2023). Experience in meditation shapes and consolidates specific neural correlates subserving different meditation styles which can alter the perception of the self. Consistent meditation practice enhances interoception which can lead to a process of self-discovery.

What is not fully known is if this process is beneficial to everyone. As mentioned above, meditation has clear reference to a number of negative experiences including autonomic hyperarousal, traumatic memory re-experiencing, and relaxation-induced panic (see Van Dam et al., 2017). Therefore, there may be detriments to some by practicing meditation to enhance interoception and those individuals might benefit from other interventions. It is also unknown if the shift between the narrative self to the experiential self is advantageous for all. More research is needed to clarify these constructs. Generally, however, at least within a healthy population, the data indicates that the process of enhancing self-awareness through meditation is associated with a number of positive outcomes which includes a process of self-discovery (see Hölzel et al., 2011; Gibson, 2014; Kok and Singer, 2017; Qin et al., 2020; Nave et al., 2021).

## Conclusion

The sense of self is complicated. There is much that is still unknown regarding meditation and the self. Spiritual traditions that include meditation in their practices provide descriptions and insights into the nature of the self. Recent advancements in neuroscience provide evidence for the neurological functions of various altered states induced by meditation. These studies also provide an additional step toward understanding the developmental process of meditation, potential benefits, and possible detriments. The primary aim of this paper was to account for the general pattern of change in the perspective of the self as one practices meditation described in the literature. The narrative self, the conceptual representation of who we think we are, appears to be heavily shaped by the external world. Many define themselves by external features such as appearance, social roles, political or religious affiliation and so forth. Maintenance of self-identifying features includes cognitive and affective factors that are consistently linked to the DMN. However, these self-referential processes associated with the DMN may not be guided by cognitive or perceptual principles *per se*, but rather the meaning or investment of self-related stimuli to maintain a fixed self-view (Northoff et al., 2006).

The experiential self, in contrast, seems to be shaped by the internal world governed by interoceptive processes that shape affective states, cognitive processes, and behavioral patterns. Meditation reliably dampens activity in the DMN and increases connectivity and activity in the insula and SN which increases interoception. Interoceptive awareness grounds us in the present moment and exerts a stabilizing influence on numerous functions, including an integrated form of self-awareness. Monti et al. (2022) wrote: “Across all levels of analysis, a common thread is the fact that the most intimate, unique, unchanging features of our selves seem to be those which are, quite literally, closest to our heart, i.e., most influenced and shaped by interoceptive signals. On the contrary, extrinsic, negotiable, transient features have a looser link with interoceptive signals. Thus, we claim that interoception provides the self-concept with a firm foundation, contributing to its stability and sanity over time by making it less permeable to external influences” (pp. 2472).

There are, however, inconsistent results found throughout the literature on the effects of meditation. In an attempt to account for some of those results, I’ve provided a rudimentary conceptual framework. Attention regulation is the basis for all meditative techniques and is believed to be the prerequisite for other beneficial mechanisms to take place. Each meditation requires a different attentional style which produces different effects and modulates different neural circuits. Van Dam et al. (2017) urged scientists and practitioners to avoid using the broad term ‘mindfulness’ and specify which meditation is being taught. In doing so, future research can tease apart important differences between various meditations and link important commonalities. This can also aid in distinguishing between mindfulness and interoception, which are related but distinct mind–body constructs. This is important because many of the benefits identified in the mindfulness and meditation literature may actually be due to increased interoception and alterations within the insula and SN (see Gibson, 2019). The insula may be the least understood and underemphasized region in the brain, yet it is the only circuit linked to all subjective experiences and modulated by all meditations (Craig, 2009; Fox and Cahn, 2021). Therefore, closer attention to this region and its rich connections throughout the brain should help illuminate some of these processes. Finally, to more fully account for the inconsistencies in the literature, I have suggested that future research incorporate early life experiences, including CT and attachment related patterns, as part of the interpretive framework. The effects of early life experience and CT have a noted effect on interoception, mindfulness, predictive coding, internal working models related to the self, and the neural circuits that underpin those functions. Including individual practitioner histories should help clarify some of the inconsistencies in the literature and potentially help identify more effective interventions for a clinical population.

Selfhood appears to be not just be embodied, but deeply embodied. Practiced meditators reveal consistent, even predictable neurological patterns associated with the DMN and SN. These subtle, yet important neuroplasticity changes are linked to increased interoception and increased interoception seems to lead to an unfolding process of self-awareness. Meister Eckhart once said: “A human being has so many skins inside, covering the depths of the heart. We know so many things, but we do not know ourselves! Why, thirty or forty skins or hides, as thick and hard as an ox’s or bear’s, cover the soul. Go into your own ground and learn to know yourself there.”
